# Cyclooxygenase-2 expression in colorectal carcinoma, adenomatous polyps and non-tumour bearing margins of resection tissues in a cohort of black Africans

**DOI:** 10.1371/journal.pone.0255235

**Published:** 2021-07-27

**Authors:** Uchenna Simon Ezenkwa, Clement Abu Okolo, Gabriel Olabiyi Ogun, Adegboyega Akere, Olufemi John Ogunbiyi

**Affiliations:** 1 Department of Pathology, University College Hospital, Ibadan, Nigeria; 2 Gastroenterology Unit, Department of Internal Medicine, University College Hospital Ibadan, Nigeria; University of North Dakota School of Medicine and Health Sciences, UNITED STATES

## Abstract

**Background:**

Emerging data suggest a negative role of cyclooxygenase-2 (COX-2) in colorectal carcinomas (CRC). Investigating this in developing communities such as ours helps to contribute to existing understanding of these lesions.

**Methods and findings:**

Formalin-fixed paraffin-embedded CRC colectomy tissues and their corresponding non-tumour margins of resected tissues were sectioned and stained with COX-2 antibody. Adenomatous polyp tissues from non-cancer bearing individuals were similarly processed for comparison. COX-2 expression was scored for percentage (< 5% = 0; 6%-25% = 1; 26%-50% = 2; 51%-75% = 3; 76%-100% = 4) and intensity (no staining = 0; yellow = 2; yellowish-brown = 3, brown = 4). Total immunoscore (percentage + intensity score) ≥ 2 was regarded as positive COX-2 expression. Outcome was statistically evaluated with clinicopathological data to determine COX-2 expression-associated and predictor variables. Ninety-five CRC cases and 27 matched non-tumour tissues as well as 31 adenomatous polyps met the inclusion criteria. Individuals with CRC had a mean age of 56.1 ± 12.6 years while those with adenomatous polyps had a median age of 65 years (range 43–88). COX-2 was differentially overexpressed in CRCs (69/95; 72.6%) and in adenomatous polyps (17/31; 54.8%) than in non-tumour tissues 5/27 (18.5%); *p* < 0.001). The difference in COX-2 expression between CRC and polyps was non-significant (*p* > 0.065). Tumour grade, advanced pT-stage, tumour-infiltrating lymphocytes, and dirty necrosis were also significantly associated with COX-2 expression (*p* < 0.035; 0.043, 0.035 and 0.004, respectively). Only dirty necrosis and Crohns-like lymphocytic aggregates predicted COX-2 expression (*p* < 0.05).

**Conclusion:**

This study showed a progressive increase in COX-2 expression from normal to adenomatous polyp and CRC tissues, this being associated with poorer prognostic indicators. Although COX-2 appears early in CRC, it may play a secondary role in promoting tumour growth and invasiveness.

## Introduction

Colorectal carcinoma (CRC) is a disease of multiple etiologies. Globally, it continues to predominate in incidence in both genders besides lung, female breast and prostate cancers [1]. Its contribution to cancer mortality is only second to lung cancer and it is projected to rise higher in developing communities [1]. Therefore, on-going efforts are directed towards reducing this trend by defining molecular features driving this disease, one of which is cyclooxygenase-2 (COX-2), an inducible enzyme with key roles in inflammation.

Emerging data have suggested possible roles for COX-2 in CRC [2]. Most of these attempts stem from observations of the effect of COX inhibitors on colorectal adenoma and cancer risk. Prostaglandin E_2_ (PGE_2_), a metabolic by-product of arachidonic acid breakdown by the COX-2 enzyme serves as a growth signaling factor in colorectal adenomas and carcinogenesis but not in normal colon tissues [3]. Inhibitors of this pathway such as aspirin have been shown to reduce the relative risk of CRC expressing COX-2. Individuals taking aspirin regularly (defined as “taking two or more standard aspirin tablets per week or using aspirin at least two times per week”) had 32% lower risk of COX-2 positive tumours compared to non-regular users. This latter group also had higher incidence of CRC [4]. Even among colorectal cancer patients on adjuvant chemotherapy following colectomies, disease-specific and overall survival with prolonged recurrence interval was shown among aspirin users compared to the control non-users [5].

Not only is COX-2 expression remarkably higher in malignant tumours than normal tissues, it is also found to be higher in larger tumours (≥ 5cm), and in tumours with increased depth of invasion (up to the serosa), poorer differentiation, advanced stage of disease (Duke stage C and D) and distant metastasis [6]. One study that reported lower COX-2 expression in CRC tissues than the adjacent normal tissues showed that the high normal-to-tumour tissue COX-2 expression correlated with high recurrence rates and poor prognosis and concluded that prostaglandin secretion by COX-2 in normal tissues could promote tumorigenesis in CRC tissue [7]. These observations support unfavorable impact on survival among CRC patients whose tumours elaborate this enzyme [8].

Studies among Africans investigating COX-2 expression in CRCs are few. Because racial differences have been noted in COX-2 expression in lung adenocarcinoma between African-Americans and Caucasians, with higher risk of death among the former [9], CRC tumour positivity with this marker hypothetically may portend worse prognosis among Africans. Adding to the established poorer CRC disease outcome in this population, this study is deemed timely as it contributes to the available knowledge on this subject.

## Materials and methods

This was a retrospective study conducted at the department of Pathology of a tertiary hospital in south-western Nigeria. Suitable formalin-fixed, paraffin-embedded (FFPE) archival tissue blocks from resected colectomy samples histologically diagnosed as harbouring colorectal carcinomas (and their non-tumour margins) were included in this study. Data such as age, gender, location of tumour within the large bowel and tumour size were obtained from the histopathology request forms. Tumours located in the caecum, ascending and proximal two-thirds of the transverse colon was documented as right large bowel while those within the distal third of the transverse colon, descending colon, sigmoid colon and the rectum were regarded as left large bowel. The archived H&E stained slides of the corresponding FFPE tissue blocks were retrieved and reviewed. Faded or missing slides were replaced with fresh ones made from the representative tissue blocks.

Carcinomas were classified according to histologic types and grades following the World Health Organization criteria [10]. Other histological features documented included tumour infiltrating lymphocytes, Crohn-like lymphocytic reaction, dirty necrosis and tumour staging. The tumour infiltrating lymphocytes was determined as presence of 2 or 3 intra-epithelial lymphocytes per 5 high power fields while Crohn-like lymphocytic reaction was determined as presence of 2 to 3 peri- or intra-tumoural lymphocytic follicle aggregates with or without a germinal center [11, 12]. Dirty necrosis was defined as the presence of tumour glandular luminal eosinophilic secretions mixed with necrotic debris and neutrophilic infiltrate [13].

Tumour staging was performed according to the TNM protocol for tumour staging [14]. The pT stage 1–4 was further categorized as pT1-pT2 and pT3-pT4 to denote tumour cells extension to the serosa (pT3-pT4) or otherwise, whilst the TNM stage I-IV was grouped as early stage (stage I-II) and late stage (III-IV) tumours.

Non-matched FFPE tissues of adenomatous polyps removed via cold polypectomy snare procedure were also retrieved and assessed for COX-2 expression. First, the H&E stained slides of these tissues were reviewed to classify them according to adenoma subtypes (tubular, villous or tubulovillous). Polyp location within the large bowel and the degree of dysplasia was also documented besides patient’s gender and age.

### Immunohistochemistry of COX-2

Cyclooxygenase-2 immunohistochemical staining was performed using the streptavidin-biotin-peroxidase method according to manufacturer’s protocol. Rabbit anti-human COX-2 antibody from Abcam laboratories (clone ab15191, Lot: 6R146689-1; Abcam, 1 Kendal Square, Cambridge, MA, USA) was used as the primary antibody.

#### Immunostaining evaluation

Immunostaining of the tumour cells and non-tumor mucosal tissue cells or lack of it was assessed semi quantitatively by estimating the percentage of cells stained and staining intensity. The percentage of cells stained was scored as follows [6]: 0 = 0%-5%; 1 = 6%-25%; 2 = 26%-50%; 3 = 51%-75%; 4 = 76%-100%. Staining intensity was scored as follows [6, 15]: 0 = no staining; 1 = faintly yellow; 2 = brownish yellow; 3 = brown. Addition of the percentage expression score (0 to 4) and staining intensity score (0 to 3) was obtained for each section examined and the outcome graded as negative (-; combined score = 0–1); weakly positive (+; combined score = 2–3); moderately positive (++; combined score = 4–5); strongly positive (+++; combined score = 6–7). For the purpose of estimating the degree of COX-2 expression in statistical analysis, the score grades were classified as negative (combined score 0–1) or positive (combined score 2–7).

### Statistical analysis

The data generated was analysed using Statistical package for social sciences tool version 20. Descriptive statistics of frequency, mean and median was used to determine proportions and central tendencies of nominal and continuous variables as appropriate. Chi-square test or Fisher exact was employed to test for associations between COX-2 expression and the biodata, tumour location, tumour size (≤5cm and >5cm) other histopathological features and association of COX-2 expression between CRC tissues, normal (non-involved margin of resection), and adenomatous polyp. Student t-test and Mann-Whitney U test was used to test for the difference in means of the continuous variables age and on tumour size respectively between COX-2 positive and negative CRC tumours. The predictive factors for COX-2 expression among all the variables were determined using binomial logistic regression analysis. Two-tailed *P* value < 0.05 was accepted as statistically significant.

### Ethical statement

This study was conducted from January 2018 to November 2018 and included colorectal carcinomas histologically diagnosed from January 2008 to December 2017. These samples were derived from those of a larger study on histomorphological and molecular characterization of tumours of the colorectum with participants’ written informed consent. Ethical approval was granted by the University of Ibadan/University College Hospital Ibadan institutional review board (UI/EC/17/0481). The data presented here was fully anonymized prior to being accessed and analyzed; as a result, the need for additional consent was waived by the institutional ethical committee.

## Results

### Clinicopathological parameters

The clinicopathological features of the patients and tumours are outlined in Table 1. Ninety-five CRC cases comprising 49 (51.6%) males and 46 (48.4) females were included in this study giving a male-to- female ratio of 1.1:1. The youngest patient was 20 years old and the oldest was 84 years old. Hence, age groups 0–9, 10–19 and above 90 years were not represented. Tumour size ranged from 1cm to 14 cm with a median value of 6cm. Eight (8.4%) of the tumours had no documented size.

**Table 1:** Clinicopathological parameters of the patients and tumour tissues.

Parameter	Frequency	Percentage
**Gender**		
Male	49	51.6
Female	46	48.4
**Tumour location**		
Colon	64	67.4
Rectum	31	32.6
**Histologic types:**		
Adenocarcinoma NOS	70	73.7
Mucinous carcinoma	20	21.1
Signet ring carcinoma	5	5.3
**Lymph node metastasis**		
Yes	37	38.9
No	47	49.5
Nx	11	11.6
**Distant tumour metastasis**		
Yes	12	12.6
No	83	87.4
**pT Stage**		
pT1	3	3.2
pT2	35	36.8
pT3	30	31.6
pT4	27	28.4
**TNM Stage**		
I	18	19
II	21	22.1
III	34	35.8
IV	12	12.6
Not Staged	10	10.5

There were 54 (56.8%) tumours located in the rectum and the left side of the colon, 30 (31.6%) on the right side while 11 (11.6%) were not localised but stated as “colon.” Rectal location accounted for 31 (32.6%) of all the tumours.

Adenocarcinoma not otherwise specified (NOS) was the commonest histologic subtype accounting for 70 (73.7%), followed by mucinous adenocarcinoma 20 (21.1%) and then signet ring cell carcinoma 5 (5.3%). Of the 70 adenocarcinomas NOS, 33 (47.1%) were well differentiated, 16 (28.6%) were moderately differentiated and 21 (24.3%) were poorly differentiated. The mucinous and signet ring carcinomas were classified as others and were not further graded.

The least occurring pT stage was pT1 with only 3 (3.2%) tumours represented in this category. Fifty-seven (60%) of the tumours had at least serosal invasion by the tumour cells. Whilst lymph node status was documented for all the tumours, eleven (11.6%) resection specimens had no lymph node found at surgical cut-up. Forty-seven (49.5%) of cases that had lymph nodes grossly were tumour negative on histology. Among the tumour-positive lymph nodes, 24 (64.9%) were in the N1 category (1–3 positive lymph nodes) whilst 13 (34.1%) were of N2 category. Twelve (12.6%) tumours had distant metastasis (M1) status.

### Adenomatous polyp features

Thirty one polyps were identified. There were more males than females (ratio of 1.2:1) with a median of 65 years (range 43–88 years). Polyp location, adenoma type and degree of dysplasia are displayed in Table 2. Nearly three-fourths of these cases were on the left side of the colon and rectum. Again about 75% were also tubular adenoma with villous and tubulovillous histology being equal in occurrences. With degree of dysplasia, there was no remarkable difference between low grade and high grade.

**Table 2:** Colorectal adenomatous polyp features and COX-2 expression by frequency.

	Frequency	Percentage	*P*
**Gender**			0.725
Male	17	54.8	
Female	14	45.2	
**Location**			0.698
Right	7	22.6	
Left	22	71.9	
Not stated	2	6.5	
**Type of adenoma**			0.600
Tubular	23	74.2	
Villous	4	12.9	
Tubulovillous	4	12.9	
**Degree of dysplasia**			0.479
Low grade	15	51.6	
High grade	16	48.4	

### Cyclooxygenase-2 expression in colorectal carcinomas

**Fig 1:**
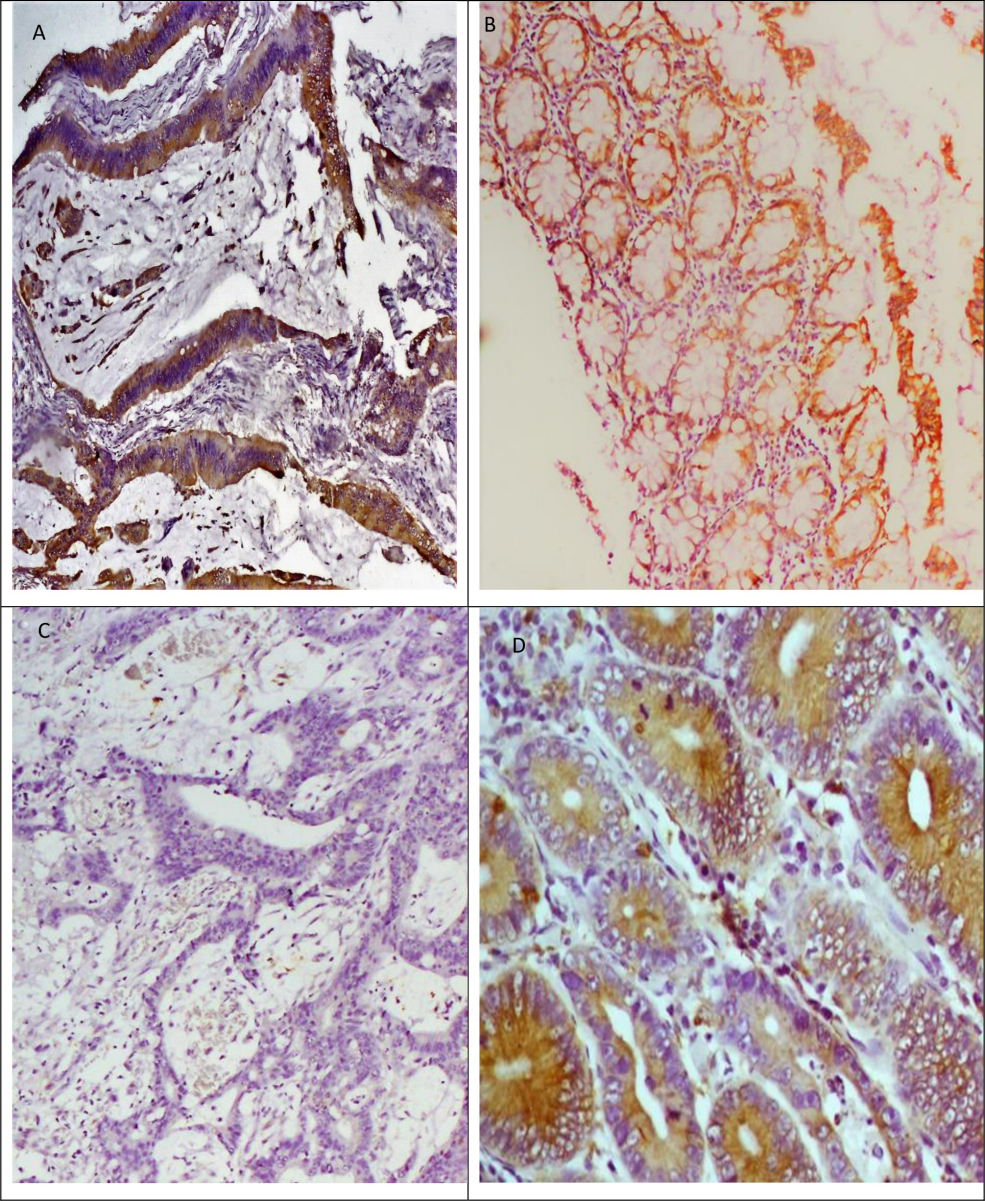
COX-2 immunohistochemical staining of colorectal tumour tissue, normal colonic mucosa from margin of resection and adenomatous polyps. In 1A, mucin is seen dissecting tissue planes with irregular malignant colorectal glands whose cytoplasm stain positive for COX-2 antibody. Normal colorectal tissue with positive COX-2 staining is shown in 1B. Fig 1C contains tumour tissue lacking COX-2 expression whereas that presented in Fig 1D represents COX-2 positive tubular adenomatous polyp tissue.

Sixty-nine (72.6%) tumours were COX-2 positive whilst 26 (27.4%) were negative. COX-2 staining was moderate (combined score 4–5) to strong (combined score 6–7) in 53 (55.8%) of the tumour tissues. Eleven (11.6%) of the remaining 16 positive tumours had a combined score of 3 whilst 5 (5.3%) had a combined score of 2. Positive and negative staining is depicted in Fig 1A and 1C.

Further statistical analysis for COX-2 expression and other clinicopathological parameters was applied on tumour tissues only. Table 3 shows the relationship between COX-2 expression and other variables. There was no remarkable difference in patient age and tumour size between COX-2 positive and negative tumours. Median tumour size was the same for both groups (6cm each; U = 732, Z = -0.23). The mean rank tumour sizes however differed little as shown in Table 3. About equal percentages of tumours in the colon and rectum showed COX-2 expression (73.4% and 71% respectively). Whilst 68% (34 out of 50) of low grade tumours showed COX-2 positivity, high grade tumours showed 91% (20 out of 22 tumours) positive staining. Similarly, tumours showing serosal invasion and beyond had higher percentage of COX-2 positivity compared with tumour extension to the muscularis propria only (80.4% vs. 61.5%). On overall TNM staging, this effect waned to about equal percentages between early (I-II) and late (III-IV) stages (71% vs. 73%). No remarkable difference was seen between tumour-positive and negative lymph nodes with regards to COX-2 expression.

**Table 3:** Relationship between COX-2 expression and some clinicopathological features of the tumours.

Variable	N	COX-2 Expression		χ^2^ value	*P*
		Positive	Negative		
**Mean Age (years)**	95	54.9 ± 17.8	56.6 ± 14.8	-	0.629^⁋^
**Mean rank tumour size**	87	44.38	43	-	0.818^٭^
**Gender**					
Male	49	36	13	0.036	0.850
Female	46	33	13		
**Tumour location**					
Colon	64	47	17	0.064	0.800
Rectum	31	22	9		
**Histology type**					
Adenocarcinoma NOS	70	52	18	0.366	0.545
Others	25	17	8		
**Tumour grade**					
Low grade	50	34	16	4.454	0.035*
High grade	22	20	2		
**pT stage**					
pT1-pT2	39	24	15	4.096	0.043*
pT3-pT4	56	45	11		
**Lymph node metastasis**					
Yes	36	27	9	0.013	0.911
No	46	34	12		
**Distant metastasis**					
Yes	12	8	4	0.246	0.730
No	83	61	22		
**TNM stage**					
I-II	38	28	10	0.040	0.842
III-IV	46	33	13		
**Crohn-like lymphocytic aggregates**					
Yes	30	20	10	0.785	0.376
No	65	49	16		
**Tumour-infiltrating lymphocytes**					
Yes	16	8	8	4.958	0.035*
No	79	61	18		
**Presence of dirty necrosis**					
Yes	28	26	2	8.170	0.004*
No	67	43	24		
**Lymphovascular permeation**					
Yes	7	6	1	0.651	0.669
No	88	63	25		
**Perineural invasion**					
Yes	14	11	3	0.291	0.751
No	81	58	23		

**N**: number of cases in each category of clincopathological parameter; **χA;**^**2**^ **value:** chi-square value; ***P***: level of significance.

* p value < 0.05

^⁋^ Student t-test statistic

٭Mann-Whitney test

Associations between COX-2 and other pathological parameters of the tumours are also shown in Table 3. High grade tumours, serosal invasion, lymph node positivity, distant metastasis, lymphovascular permeation and perineural invasion showed consistent higher COX-2 expression (90.5%, 68.2%, 75%, 66.7%, 85.7%, and 75% respectively). Of all these parameters, tumour grade and serosal invasion showed significant association with COX-2 expression (p < 0.035; and 0.043 respectively). Other parameters significantly associated with COX-expression were tumour infiltrating lymphocytes (TIL), and presence of dirty necrosis (Table 3). Eight (50%) of the tumours with TIL showed negative COX-2 expression and 6 (75%) of these were in the pT1-pT2 category.

Predictors of COX-2 expression in CRC were significant for presence of dirty necrosis and Crohn-like lymphocytic aggregates (Table 4). The odds for COX-2 positivity increases by a factor of 11.3 for each CLA-positive tumour, while that for dirty necrosis diminishes by a factor of 0.006. Lymphovascular permeation, tumour grade and histological subtype showed increased likelihood of COX-2 positivity as shown by their higher odds ratio, although, this remained non-significant (Table 4). Age, gender and tumour size also had no significant effect on COX-2 expression by tumour cells.

**Table 4:** Predictors of COX-2 expression among the clinicopathological parameters.

Variable	Odds ratio	95% CI	*P*
Age	1.027	0.975–1.08	0.319
Gender	1.108	0.213–5.76	0.903
Tumour size	0.777	0.549–1.10	0.156
Tumour location	3.270	0.490–21.80	0.221
Histologic tumour subtype	5.071	0.325–79.21	0.247
Tumour grade	19.267	0.752–493.89	0.074
pT stage	0.786	0.156–3.94	0.770
Lymph node status	0.894	0.169–4.71	0.895
Metastasis	0.915	0.115–7.30	0.933
Lymphovascular invasion	29.477	0.556–1563.0	0.095
Perineural invasion	2.072	0.108–39.76	0.629
TIL	2.272	0.357–14.46	0.385
CLA	11.312	1.652–77.45	0.013*
Dirty necrosis	0.006	0.000–0.21	0.005*

CI = confidence interval; TIL: Tumour-infiltrating lymphocytes; CRA: Crohn-like lymphocytic aggregates.

*P < 0.*05*

The photomicrographs of the immunostaining of the CRC tumours are as shown in Fig 1.

### Colorectal carcinoma, margin of resection and adenomatous polyps

Twenty seven margins of resection tissues matched with their corresponding colectomy specimen margins of resection were found and included in this study. The remaining colectomy specimens had missing FFPE tissue blocks margins of resection. These margins of resection tissues showed positive COX-2 expression in 5 (18.5%) of the 27 tissues. Only one of these five tissues showed strong positivity with combined score of 6 (Fig 1B). Interestingly the corresponding tumour tissue had a combined score of 3. The other 4 COX-2 positive margins of resection tissues each had combined scores of 2. Six out of the 22 margins of resection tissues with negative COX-2 expression had their corresponding tumour tissues staining negative. The remaining 16 (72.7%) margins of resection tissues with negative COX-2 expression had positive COX-2 expression in their corresponding CRC tumour tissues.

Adenomas had slight difference in COX-2 positivity, being positive in 17 (54.8%) tissues. Combined score in these cases were mostly high, 10 out of 17 positives had a combined score of 4 and above. A representative positive tissue is shown in Fig 1D. Of the negative polyps, all had score 0.

Association of COX-2 expression with CRC, normal (margin of resection) and polyp tissues are as shown in Table 5. Whereas the difference between CRC and normal tissues and that between adenomas and normal tissues where significant, that between CRC and adenomas was not.

**Table 5:** Crosstabulation of COX-2expression between CRC and uninvolved resection margin, adenomatous polyps and uninvolved resection margin, and CRC and adenomatous polyp.

Variable	N	COX-2 Expression		χ2 value	*P value*
		Yes	No		
**CRC**	95	69	26	54.989	< 0.001*
**Normal Tissue**	27	5	22		
**Adenoma**	31	17	14	167.386	< 0.001*
**Normal Tissue**	27	5	22		
**CRC**	95	69	26	3.415	0.065
**Adenoma**	31	17	14		

*p < 0.001

## Discussion

The demographic pattern of CRC in this population has not changed remarkably over recent years. The nearly equal male-female ratio seen presently is similar to earlier reports by Irabor *et al*, [16] in Ibadan and Abdulkareem *et al* [17], in Lagos Nigeria. More tumours still occur in the left colon and rectum with advanced stage at presentation [16–18]. However, we have documented a higher mean age of 56.1 years compared to 41 years and 50.7 years documented previously by Irabor and Abdulkareem respectively, both being among populations within the same geographic location of the country as the present study [16, 17]. This difference in age could be due to study population selection bias. Whereas the present study involved only individuals who underwent surgical resection of their tumours, that by Irabor *et al* [16] and Abdulkareem *et al* [17] did not discriminate between type of tissue specimens and may be more representative. Overall, age of occurrence is lower when compared with studies from elsewhere such as the United States of America with mean age above 60 years [19]. Screening programs and health education to discourage sedentary and detrimental dietary lifestyles are urgently needed.

We show here that COX-2, an inducible inflammatory enzyme is increasingly expressed in CRC tissues from our patients whereas the non-tumour margins of resection remain largely repressed. Seventy-three percent (69) of the tumours in this study showed COX-2 expression. The result is similar to what was reported in the study by Wu and Sun among the Chinese, who found a positive COX-2 expression of 78% [6]. It is however higher than that reported in the studies by Lim *et al* [20] and Chan *et al* [4] who showed positive expression rates of 48% and 67% among Korean and North America patients respectively. These differences might be a result of varying methods of scoring used in the different studies. Whilst this study used a combined score of ≥ 2, Lim *et al* adopted a combined score of ≥ 3 as positive whereas Chan *et al* used only intensity of staining score. In addition, there is a significant difference in COX-2 expression between tumour tissue and normal colonic tissue with normal tissue showing generally low expression of COX-2. Whilst this study showed 18.5% expression of COX-2 in normal tissue, that by Wu *et al* [6] had shown 12% COX-2 expression. This slight difference may possibly have something to do with the distance between the tumour and the normal margin examined. Wu *et al* [6] had used adjacent non-tumour tissue at a distance of 5cm and above away from the tumour as control but the exact distances beyond 5cm from the tumour was not taken into account in that study. Our study used colorectal tissues from the margins of resection. The distances away from the tumour are also bound to vary with colectomy lengths. Notwithstanding, both studies, found a significant difference in COX-2 expression between CRC and normal colonic tissues, which has also been demonstrated at the molecular level with significant difference in COX-2 mRNA expression between CRC and normal colon tissue [21, 22].

COX-2 is known to synthesize PGE_2_ which serves as a growth signaling factor in colorectal adenomas and carcinogenesis but not in normal colon tissues [3]. This may therefore account for the significant difference in expression between CRC and normal tissues, adenomas and normal mucosa but not between CRC and adenomas. In addition, PGE_2_-associated transformation from adenoma to malignancy is believed to be dose-dependent [3]. Thus, whereas CRC had 72.5% expression, adenomas had 54.8% while non-tumour tissues had 18.5% positivity in the present study, suggesting cumulative effect of increasing doses of the enzyme as carcinogenesis progresses. Further to this, a recent study by Wang *et al*, demonstrated that individuals with colorectal adenomas expressing elevated COX-2 prior to treatment with celecoxib, a COX-2 inhibitor, were more likely to have fewer adenomas at follow-up surveillance compared to low COX-2-expressing adenoma patients [23]. Thus, CRC with high marker expression will be expected to respond more favorably to anti-COX-2 therapies.

Positive COX-2 expression by adjacent non-tumour colorectal mucosa may represent a transitioning from a mucosa-at-risk of developing malignancy to a mucosa that has evolved to malignancy. It may also suggest an inductive effect of the tumour microenvironment on the adjacent non-tumour mucosa [24] Lin *et al* [7] in their study found a higher and more intense COX-2 expression by normal colon tissue compared to CRC tissues and hypothesized that the COX-2 expression by normal colonic mucosa adjacent to the tumour tissues modifies the tumour-stromal microenvironment thereby promoting tumourigenesis. Since fewer of the margins of resection in our data had positive COX-2 status; we are not able to substantiate this position. Defining a cut-off point for the distance away from the tumour in which COX-2 is expressed, may shed more light in this regard.

Another important finding was that high tumour grade and late tumour pT stage were significantly associated with COX-2 expression by the tumours. This is similar to the study by Wu *et al* [6] Lim *et al* [20] and Roelofs *et al* [21] who also found significant association between COX-2 expression and tumour pT stage but not with tumour grade. The consistent association between pT stage and COX-2 expression seen in all these studies suggests a role for COX-2 in tumour cell invasiveness. Most of the CRC tumours in these studies, as in the present study, had tumour invasion up to the serosal layer [6, 20, 21].

Observed predominance of COX-2 overexpression in tumours displaying lymphovascular permeation, lymph node metastasis, distant metastasis and late TNM stage in this study supports a role of COX-2 in enhancing tumour spread, as has been shown by Zhu *et al* [25]. This is similar to the studies by Lim *et al*, Roelofs *et al*, and Wu *et al* although only Wu *et al* found significant association between COX-2 and all these parameters [6, 20, 26]. Our study sample size is similar to those by Lim *et al* [20] and Roelofs *et al* [26] which is smaller compared to the larger population in the study by Wu *et al* [6]. Notwithstanding, the consistent higher expression of COX-2 in tumours with these poorer prognostic factors may indicate that the role of COX-2 in tumour progression persists beyond mucosal epithelial transformation to adenoma and thence to carcinoma.

The significant association between TIL and COX-2 expression in this study suggests a role for COX-2 in lymphocyte immune response to CRC. Credit to this assertion is the finding that majority (75%) of the COX-2 negative tumours had a low PT stage (pT1-pT2) whereas higher pT stage was associated with increased COX-2 positivity. Data describing role of COX-2 expression in modulating immune responses in various cancers are emerging [27–29]. Investigators have shown that PGE_2_ produced by COX-2 induces local immunosuppression by suppressing Dendritic cells (DCs), natural killer (NK), T cells, type-1 immunity excluding type-2 immunity which promote tumor immune evasioninhibiting [30]. Wang *et al* [27] described an experiment suggesting that COX-2 could act in an autocrine manner to regulate FasL and TRAIL expression by CRC cells that causes cytotoxic T-lymphocytes and NK cell apoptosis. This leads to “compromised host immune responses to tumour” [27]. Another proposed mechanism is the selective recruitment of regulatory T-cells by COX-2-expressing tumours thereby modulating anti-tumour immune responses [28]. This is akin to “immunoediting” described in the study by Zelenay *et al* [29]. These authors showed that Braf^v600E^ melanoma cells deficient in PGE_2_ synthase-1 and 2 were spontaneously rejected in immune competent recipient mice but survived and grew progressively in T- and B- cell-deficient ones [29]. PGE_2_ therefore, modulates anti-tumour immune activity and permits tumour progression.

The other proposed prognostic feature is dirty necrosis. In this study, dirty necrosis was strongly associated with COX-2 expression. It strongly predicted COX-2 expression in tumour cells. It is not apparently clear what link this might have on the tumour biology. Dirty necrosis is more commonly seen in microsatellite stable CRC tumours [13]. Luminal eosinophilic secretions within CRC tumour glands stain differently to PAS, MUC1 and MUC2, suggesting differences in tumour biology [13, 31]. Tumour glandular luminal eosinophilic secretions containing necrotic debris (dirty necrosis) stain positive to PAS and MUC1 positive whereas those without necrotic debris stain MUC2 positive [13, 31] It could therefore be suggested that CRCs harboring dirty necrosis may have a molecular characteristic different from others which might influence or be influenced by COX-2 expressivity. This observation in this study requires further studies to elucidate or refute its relevance in colorectal carcinogenesis.

In all, the result of logistic regression statistics in this study showed that only Crohn-like lymphocytic aggregates (CLA) and dirty necrosis predict COX-2 expression independently in CRC tissues reliably. COX-2 expression may therefore represent an acquired phenotype that modulates tumour immunity irrespective of other prognostic clinicopathological features. How this characteristic influences tumour stage and differentiation alongside the presence of dirty necrosis requires further investigation.

A study describing CLA, TIL, and dirty necrosis in CRC from this environment found a frequency of 43%, 48% and 20% each for CRA, TIL and dirty necrosis respectively and these figures are slightly higher than the results of this present study [32].

This present study documented CRC tumour size in this environment, a feature not commonly observed in previous studies. Tumour size distribution in this study is similar to those of the study by Mehmet and Yasemin among the Turkish population with size ranging from 1cm to 15 cm [33]. Although tumour size is not a component of tumour staging, studies have found significant association between tumour size and worse patient survival [34]. Its documentation may also provide information on how large tumours tend to grow prior to diagnosis in a given population.

Whereas expression of COX-2 has been suggested to influence CRC tumour size in a study by Fujita et al among the Japanese [35], the present study did not reach similar conclusions. However, COX-2 expressing tumours in our study had slightly higher tumour size than non-expressing tumours. Remarkable differences in COX-2 estimation exist between our studies and that by Fujita *et al*. Firstly, whilst we assessed only COX-2 and its effect on tumour size, Fujita *et al* used COX-2 index which was defined as the ratio of COX-2 to COX-1 (COX-2/COX-1) in the colorectal carcinomas. Secondly, tumour sizes among the Japanese were grouped into three classes, (≤3cm, ≤ 6cm, and > 6cm), in contrast to two categorizations used in the index study. To what extent these observed differences might influence the data in both studies is not certain presently. Subsequent study design to investigate this hypothesis is needed to elucidate this further.

Taken together, available data supports a worse prognosis in CRC patients whose tumours overexpress the marker, but how this is brought about is yet to be fully elucidated. A causal role has been suggested [36]. Earlier studies investigating COX-2expression in non-neoplastic, adenomatous and cancer cells from the colon has reached similar findings of increasing marker expression from normal to adenomas and carcinoma tissues as the present study [37, 38]. Whether this represents an early event in colorectal carcinogenesis [37], remains to be proven.

Yet other studies have suggested a secondary effect of COX-2 expression in promoting development of CRC. Stromal fibroblast within tumor microenvironment has been shown to elaborate COX-2 [2]. In carcinoma cells, this expression is observed within the cytoplasm of the epithelial cells [2]. COX-2 is known to induce angiogenesis by elaborating vascular endothelial growth factor [39]. Another study reported increased cell proliferation and invasiveness of CRC cells bearing overexpression of COX-2 [25]. These studies also showed early detection of COX-2 in these tumours [25, 39] and could contribute to higher tumour size [35]. In effect, angiogenic potential of COX-2 mediators supports tumour growth leading to increase in tumour size and provides vascular network for tumour metastasis.

### Limitations to the study

Lack of survival (follow up) data for this study population is a major limitation of this study since COX-2 expression has been shown to predict poorer outcome in CRC patients [6]. Survival data is generally difficult to come by in this environment as this has not been a major focus of data collection up until very recently [40].

Secondly, as COX-2 inhibitors could affect COX-2 expression significantly in CRC tissues [4], knowledge of COX-2 inhibitor use by the patients would be helpful in further interpretation of these results. Despite this, our findings regarding COX-2 expression did not differ appreciably from the studies that evaluated the effect of aspirin intake on COX-2 expression in CRCs [4, 6]. Therefore, the results of this present study are likely a true reflection of COX-2 expression status in the population studied and that this observation is similar to CRC tumours elsewhere.

## Conclusion

Cyclooxygenase-2 in CRC is associated with poorer tumour indices of high tumour grade and stage (tumour invasion), and could influence tumour immunity in the hosts either by modulating lymphocytic response to tumour cells or by recruiting T-regulatory lymphocytes thus promoting tumour progression. This effect is seen as the mucosa transitions from normal to carcinoma tissues, with intermediate expression in colorectal adenomas. Further studies are required to verify a primary or secondary role of this marker in CRC. COX-2 being a drug target can also offer insights into synergistic control of this disease.

## Supporting information

S1 Dataset (XLSX)
